# Substance use disorder risk assessment: positive emotional experiences with first time use and substance use disorder risk

**DOI:** 10.3389/fpsyt.2024.1368598

**Published:** 2024-09-20

**Authors:** Karen E. Arscott, Donna M. Eget, Maria C. Marcos, Brian J. Piper

**Affiliations:** ^1^ Geisinger Commonwealth School of Medicine, Geisinger Health System, Scranton, PA, United States; ^2^ Medicus Urgent Care, Dunmore, PA, United States; ^3^ Center for Pharmacy Innovation and Outcomes, Genomics and Autism & Developmental Medicine Institute, Geisinger Precision Health Center, Forty Fort, PA, United States

**Keywords:** alcohol use disorder, screening, emotion, medication assisted treatment, Preaddiction, prevention

## Abstract

**Introduction:**

Substance Use Disorder (SUD) screening tools used in current practice are designed to identify SUD once patients have begun regular dangerous drug use. While these screening tools are valuable, prevention and avoidance of SUD would save countless lives. The climbing number of deaths due to drug overdose make screening for and prevention of SUD imperative. This study addresses this care gap. The aim was to develop a simple screening tool for patients who may be prone to develop Alcohol Use Disorder (AUD) and/or SUD prior to addiction. It was hypothesized that participants with initially positive emotional experiences would be correlated with a future SUD diagnosis.

**Methods:**

The study involved a self-administered survey using a cross-sectional design and was carried out over one-month in the spring of 2021. Those patients who presented to the MAT clinic (SUD group) were seen in a separate area than the patients presenting for urgent care (Comparison group). Participants (N = 259) were voluntarily recruited from MAT and Urgent care: Patients receiving acute care were assigned to the Comparison (N = 126, 50.8% female, 5.7% non-white, 27.2% age < 34) and those receiving treatment for SUD were assigned to the MAT group (N =133, 40.8% female, 4.8% non-white, 36.8% ≤34). The survey questioned demographics (4 items), risk factors for AUD/SUD (6 items), information about first alcohol/opioid experiences (16 items), and factors for seeking AUD/SUD treatment and recovery (2 items). Feelings were categorized as positive (e.g., euphoria, happiness, self-confident), neutral (e.g., nothing, normal), or negative (e.g., depressed, sad, sick).

**Results:**

The MAT group felt more positive feelings with first usage of alcohol and opioids compared to the comparison group (p<.001). With first usage of opioids specifically, MAT (0.13 ± 0.04) and comparison (0.29 ± 0.07) groups differed (p <.001). Over half (55.3%), of the MAT participants reported feeling self-confident with first use of alcohol while only 29.7% of the comparison reported this (p<.001). Over three-fifths (63.7%) of the MAT group reported feeling of euphoria with the first usage of opioids compared to one-tenth (9.8%) in the comparison group (p<.001).

**Discussion:**

This retrospective cross-sectional report shows the first affective responses to substances may predict risk for future SUD and could be a prevention screening tool. Asking patients about positive feelings with first usage of alcohol/opioids could be a simple screening tool employed for prevention.

## Introduction

The current screening tools used to determine if a person has an AUD or SUD are unfortunately designed to discover these diseases at a late stage. As with most diseases, the best method would be to prevent AUD or SUD. To date, there is no research describing a tool such as this study proposes. A study conducted in 2016 examined the question of whether there is a difference between prescribed opioids or those taken experimentally and provided a starting point for investigating the feelings persons experience the first time an opioid is used (1). This “initial experience” is the basis for this research project. Tools currently in use for AUD are repurposed for other substances. These tools: CAGE (Acronym Questionnaire) (2); Michigan Alcohol Screening Test (MAST) (3, 4); Alcohol Use Disorder Identification Test (AUDIT) (5); Current Opioid Misuse Measure (COMM) (6); Rapid Opioid Dependence Screen (RODS) (7); Leeds Dependence Questionnaire (LDQ) (8); Tobacco, Alcohol, Prescription medications and other Substance (TAPS) Tool (9); Opioid Risk Tool (ORT) (10); and Alcohol Screening and Brief Intervention (ASBI) (11) are all designed to identify an AUD/SUD after it is an addiction and a serious problem.

The purpose of this study was to uncover an earlier point of identification for prevention – perhaps as a “Preaddiction” flag as discussed elsewhere (12). While working at a Medical Assisted Treatment (MAT) clinic, many patients with AUD/SUD described their initial experience with alcohol or opioids as “great”, “best I have ever felt”, “finally felt normal”, “amazing”, etc. These descriptions are different than many other persons who have described their initial experience as “nauseating, terrible, or no help with pain.” The hypothesis was that persons with potential for AUD and/or SUD have a predisposition that can be determined simply by asking the question how they felt with their initial substance experience.

## Materials and methods

### Participants

Potential study participants (N=259) were identified at a local MAT clinic and urgent care facility during their standard care visits in either the MAT clinic or as persons requiring another type of medical care at the urgent care. This MAT clinic supports patients seeking care for alcohol and/or opioid use disorders. Patients receiving acute care were assigned to the Comparison group (N = 126, 50.8% female, 5.7% non-white, 27.2% age ≤ 34) and those receiving treatment for SUD were assigned to the Medical Assisted Treatment (MAT) group (N =133, 40.8% female, 4.8% non-White, 36.8% age ≤ 34). Participants were recruited over the month of May 2021.

### Study design and setting

This study involved a self-administered survey using a cross-sectional design. This study was approved by the Geisinger Institutional Review Board. The researchers used a simple group of questions administered to both the SUD group (those with known SUD) and the Comparison group (persons presenting to the clinic for treatment other than SUD). Those patients that present to the MAT Clinic (SUD group) were seen in a separate area than the patients coming for urgent care (Comparison group), and therefore the respective surveys were presented at the two different areas within the clinic.

The introductory description included an Information Sheet describing the study and containing all elements of an informed consent form. The data collected was non-identifiable. The Information Sheet also included a return phone number to call should the subject have any questions or wish to withdraw from the study. Each participant was provided a unique study number for the subject to reference when requesting their study data be removed from the study. Surveys included questions about demographics (four items), risk factors for AUD/SUD (six items), information about their first alcohol and opioid experiences (sixteen items), and factors for seeking AUD/SUD treatment and recovery (two items). There were items about first-time usage and participants were asked to select the emotions that they experienced. Feelings were categorized as positive (e.g., euphoria, happiness, self-confident), neutral (e.g., nothing, normal), or negative (e.g., depressed, sad, sick).

### Data-analysis

Responses were collected and entered into *Systat*, version 13.1 for analysis. Comparisons between the MAT and Comparison groups were made with t-test for parametric variables, with Levene’s test employed to assess that the homogeneity of variance assumption was met, and chi-square or Fisher’s exact test (if N/cell ≤ 5) for non-parametric variables. A p <.05 was considered statistically significant although statistics that met a more conservative alpha (e.g. p <.001) or trends (p >.05 but p <.10) were noted. Significant differences on parametric variables were expressed as Cohen’s *d* with 0.2, 0.5, and 0.8 interpreted as small, medium, and large (respectively) effect sizes.

## Results

### Participant demographics and personal medical history

Over half (52.4%) of the 133 person MAT group had less than or the equivalent than a high school education, significantly higher than the Comparison group (31.6%, p <.001). Almost a third (32.3%) of the MAT group reported that they were not content with their current situation (p <.001). Participants in the MAT group reported incidence of family history of AUD, illegal SUD, and prescription SUD that was significantly higher than the Comparison group. The MAT group reported significantly increased personal history of AUD, illegal SUD, and prescription SUD compared to the Comparison group (p <.001). A third (32.5%) of the MAT participants stated that they had a forced sexual experience in childhood (p <.001). A subset (19.8%) of the Comparison group and 22.3% of the MAT group reported a personal history of ADD, OCD, bipolar, and schizophrenia. Over two-fifths (43.5%) of the MAT group and one-third (32.8%) of the Comparison group had a history of depression (**Table 1**).

**Table 1:** Demographics and history of medication assisted treatment (MAT N=133) and comparison (N=126) groups.

Question	Comparison Group	MAT Group	P value
Female (%)	50.8	40.8	
Age (%<34)	36.8	27.2	
Race (% non-white)	5.7	4.8	
Education (%<high school)	31.6	52.4	<.001
Content with current situation (% no)	9.2	32.2	<.001
FH AUD (% yes)	37.9	53.6	<.05
FH of illegal SUD (% yes)	21.8	44.4	<.001
Personal history of AUD (% yes)	18.2	60.0	<.001
Personal history of illegal SUD (% yes)	7.6	80.0	<.001
Personal history of prescription SUD (%yes)	7.6	80.0	<.001
Depression (% yes)	32.8	43.5	<.001
Forced sexual experience	8.3	32.5	<.001
ADD, OCD, Bipolar, Schizophrenia (% yes)	19.8	22.3	

ADD, Attention Deficit Disorder; AUD, Alcohol Use Disorder; FH, Family History; OCD, Obsessive Compulsive Disorder; SUD, Substance Use Disorder.

The MAT group felt more positive feelings with first usage of alcohol and opioids compared to the Comparison group (p <.001). With first usage of opioids specifically, MAT (0.13 ± 0.04) and Comparison (0.29 ± 0.07, p <.001, *d* = 2.81) groups differed. Over half (55.3%), of the MAT participants reported feeling self-confident with first use of alcohol while only 29.7% of the Comparison reported this (p <.001). The majority (63.7%) of the MAT group reported feeling euphoria with the first usage of opioids compared to 9.8% in the Comparison group (p <.001).

### Opioid usage and feelings with first exposure

The age of first opioid use was not significant between the Comparison group and the MAT group. In the Comparison group, all the participants reported taking a non-heroin opioid during their first use. Specifically with first opioid use, over half (53.7%) of the MAT group were given their first opioid from a non-provider (i.e., friend, family member) and 15.9% of the Comparison group received their first opioid from a non-provider (p <.001).

The MAT group felt more positive feelings with first opioid use compared to the Comparison group (p <.001, *d* = 2.28). Except for feeling good enough, all the positive classified feelings were significantly increased in the MAT group (**Table 2**). For example, almost two-thirds (63.7%) of the MAT group reported feeling euphoria with the first usage of opioids compared to 9.8% in the Comparison group (p <.001).

Furthermore, there was a significant difference in overall negative feelings with first opioid usage MAT participants (0.13 ± 0.04) and Comparison participants (0.29 ± 0.07, p <.001, *d* = 0.53). However, there was not a significant difference shown with comparing individual feelings that were classified as negative.

There was not a significant difference in reported neutral feelings with first time use in both groups. Over a quarter (28.6%) of the MAT group felt numb with their first opioid use, while 11.3% of the Comparison group reported this feeling (p <.001). A fifth (19.8%) reported feeling normal in the MAT group and 8.3% of the Comparison group described feeling normal with first opioid use (p <.05). There was a nonsignificant difference between the MAT group (4.0%) and Comparison group (8.3%) when reporting feeling nothing (**Table 2**).

**Table 2:** Opioid use and feelings in the medication assisted treatment (MAT, N=133) and comparison (N=126) groups.

Question	Comparison Group	MAT Group	P Value
Age of first opioid use <15 (%)	4.7	21.6	
Age of first opioid use >15 (%)	95.3	78.4	
Type: Heroin (%)	0	15.8	<.001
Source: Provider (%)	84.1	46.3	<.001
Feelings			
Positive (mean + SD)	1.30 (1.06)	3.04 (0.20)	<.001
Euphoria (%)	9.8	63.7	<.001
Happiness (%)	8.3	60.3	<.001
Self-Confident (%)	3.8	41.3	<.001
Well-Being (%)	3.0	23.0	<.001
Relief (%)	23.3	42.9	<.001
Accepted (%)	0	13.5	<.001
Strong (%)	0.8	20.6	<.001
Loveable (%)	1.5	7.9	<.05
Good enough (%)	6.0	7.1	
Focused (%)	2.3	19.8	<.001
Negative (mean + SD)	0.29 (0.07)	0.13 (0.42)	<.001
Weak (%)	3.0	0.8	
Sick (%)	9.8	8.7	
Depressed (%)	0.0	1.6	
Sad (%)	0.8	1.6	
Neutral (mean + SD)	0.06 (0.56)	0.53 (0.72)	
Normal (%)	8.3	19.8	<.05
Numb (%)	11.3	28.6	<.001
Nothing (%)	8.3	4.0	

SD, Standard Deviation.

### Alcohol usage and feelings with first exposure

In evaluating the MAT and Comparison group, 14.5% of the MAT group had taken alcohol younger than the age of 10 compared to 0.8% of the Comparison group (p <.001). There was not a significant difference in alcohol type and source between both groups.

The MAT group overall felt more positive feelings with first usage of alcohol compared to the Comparison group (p <.001, *d* = .57). Euphoria was experienced in 39.0% of the MAT group and in 12.5% of the Comparison group (p <.001). Over half (55.3%) of the MAT participants reported feeling self-confident with first use of alcohol while only a little over one-quarter (29.7%) of the Comparison group reported this feeling (p <.001). Feelings of happiness and well-being were significantly higher (p <.05) in the MAT group than in the Comparison group. Feelings of relief, being accepted, strong, loveable, good enough, and focused were not experienced in either group with first time use (**Table 3**).

**Table 3:** Alcohol use and feelings in the medication assisted treatment (MAT, N=133) and comparison (N=126) groups.

Question	Comparison Group	MAT Group	P Value
Age at first alcohol <10 (% yes)	0.8	14.5	<.001
Age of First Alcohol <15 (% yes)	30.8	60.5	<.001
Alcohol Type -beer (%)	67.7	72.6	
Alcohol type – liquor (%)	67.7	72.6	
Alcohol Source – friend (%)	49.6	42.7	
Alcohol Source – Self (%)	34.9	44.4	
Alcohol Source – parent (%)	7.0	4.0	
Feelings			
Positive (mean + SD)	0.95 (1.02)	1.63 (1.35)	<.001
Euphoria (%)	12.5	39.0	<.001
Happiness (%)	40.6	55.3	<.05
Self-Confident (%)	29.7	47.2	<.001
Well-Being (%)	11.7	21.2	<.05
Negative (mean + SD)	0.27 (0.57)	0.42 (0.76)	.078
Weak (%)	0	0	
Sick (%)	21.1	26.8	
Depressed (%)	1.6	7.3	.055
Sad (%)	3.9	7.3	
Neutral (mean + SD)	0.61 (0.63)	0.42 (0.60)	<.05
Normal (%)	31.2	19.5	<.05
Nothing (%)	22.8	29.7	

SD, Standard Deviation.

There was not a significant difference in reported negative feelings with first time use (p = .078).

Participants in the Comparison group (0.61 ± 0.06) encountered more neutral feelings associated with first alcohol use than the MAT group (0.42 ± 0.05, p <.05, *d* = .30). Almost one-third (31.3%) of the Comparison group endorsed feeling normal, while only one-fifth (19.5%) of the MAT group reported this feeling (p <.05). There was a negligible difference between the MAT group (29.7%) and Comparison group (22.8%) when reporting feeling nothing (**Table 3**).

## Discussion

This novel retrospective cross-sectional study determined that the first affective responses to recreational drug use may predict risk for future drug misuse potentially leading to SUD. Reporting positive feelings with first usage of alcohol and/or opioids could be used as a screening tool for patients who may be more prone to developing AUD and/or SUD. It is possible that the reported negative life experiences accentuated the positive experience in the MAT group – resulting in a participant using the substance excessively for that positive experience. Group differences were generally less pronounced for neutral or negative feelings. This is an important methodological development which overcomes limitations with past instruments (2–11).

The possibility of a patient with a SUD presenting to the Urgent Care and thus part of the Comparison group was considered and corrected for by asking identical questions (#12, #13, and #14) covering potential co-occurring use disorders in the survey. The number of participants in the Comparison group responding positive to a personal history of SUD or AUD is not significant.

There were over 100,000 deaths in 2021 in the US from drug overdoses (13). Similarly, there were over 140,000 deaths per year in the US due to excessive alcohol use (14). Although there are many FDA approved pharmacotherapies for AUD and opioid use disorder (OUD), addiction is a relapsing and remitting disease. There are substantial individual differences in treatment response. For example, the number needed to prevent a return to drinking was twelve for acamprosate and twelve for naltrexone (15). Prevention of AUD and SUD, perhaps using instruments like that described in this report, should be a pressing public health priority.

The age of first use for alcohol was younger in the MAT group as opposed to the Comparison group. It has been established that the younger age of exposure to substances is a risk factor for developing a SUD. This may have contributed to our findings at least in part (16).

Another interesting area is in sources of alcohol or opioids for first time use. The sources of alcohol provided are represented in **Table 3** and these are not statistically significant. However, for opioid use there is statistical significance for first time use with the substance offered by a provider (84.1% Comparison group to 46.3% for MAT group). These percentages demonstrate many patients receiving opioids from a provider for the first use. This is the point when a discussion concerning risks could be initiated by the provider – and in particular the aspect of this study and positive feelings with first use.

Some caveats and limitations are noteworthy. Although our sample size (N = 259) was sufficient to detect statistical significance across multiple areas, an increased number of participants may have strengthened this report. Not all findings were hypothesized *a priori* and although statistics that met a more conservative alpha were noted, no corrections were made for multiple comparisons. In addition, we made the decision to consider “normal” as a neutral feeling. If we had worded the question differently, this may have fallen on the positive side. If a participant felt “abnormal” and the use of a substance allowed the feeling of “normalcy” it would be positive. Future research will be necessary to further refine this instrument including with a more diverse sample (e.g., non-English speakers).

## Conclusions

The primary goal of this retrospective cross-sectional study was to discover a simple screening tool for AUD/SUD prevention. It is understood that the natural reward system is activated in persons by certain substances. When the reward pathways are activated the person experiences a pleasing or positive feeling. In the DSM-5 it is proposed that certain individuals may be “particularly predisposed” to develop a SUD (17). The defined hypothesis was supported by the data collected and analyzed (**Table 4**). With mortality from the opioid crisis escalating despite MAT (13) and a wide selection of screening tools (2–11), it is evident that prevention of the disease is required to change the trajectory of morbidity and mortality. We are cautiously optimistic that novel instruments to uncover a “Preaddiction state” (12) could contribute to precision medicine. Whenever a patient is prescribed an opioid for any reason and at any age the initial “feeling” that patient experiences should be noted. If their initial general mood is one of euphoria, confidence, or contentment, that individual should be cautioned about the potential for developing a substance use disorder if the patient begins taking the prescribed medication for the positive feeling and not for the intended pain management. This point could and should be discussed at follow-up visits and the prescribed opioid length adjusted appropriately as the acute pain is limited and the life stressors often continue. It could be documented as a positive “Preaddiction” screen and so careful prescribing of addicting medications would be warranted. Positive screening should not limit the use of potentially addictive medications if such medication is indicated. This screening tool could be widely shared in media, schools, medical/surgical offices, dental offices, and pediatric offices as a warning. Additional research in this area is warranted.

**Table 4:** Alcohol and opioid feelings summative in the medication assisted treatment (MAT, N=133) and comparison (N=126) groups.

Feelings	Alcohol	Opioid	P Value
MAT Positive (Mean + SD	1.64 (2.05)	3.09 (2.05)	<.001
MAT Negative (Mean + SD)	0.42 (0.83)	0.12 (0.83)	<.001
MAT Neutral (Mean + SD)	0.41 (0.82)	0.54 ().82)	0.08
Comparison Positive (mean + SD)	1.02 (1.31)	1.26 (1.31)	
Comparison Negative (mean + SD)	0.34 (0.64)	0.30 (0.64)	
Comparison Neutral (Mean + SD)	0.48 (0.81)	0.61 (0.81)	

SD, Standard Deviation.

## Data Availability

The raw data supporting the conclusions of this article will be made available by the authors, without undue reservation.

## Supplementary material

Supplementary Data Sheet 1 Comparison group survey.

Supplementary Data Sheet 2 Medical assisted treatment clinic group survey.
