# Supplementary material for: Substance use disorder risk assessment: positive emotional experiences with first time use and substance use disorder risk
Source: Front Psychiatry. 2024 Sep 20;15:1368598. doi: 10.3389/fpsyt.2024.1368598 (PMC11451412; doi:10.3389/fpsyt.2024.1368598)
Supplement: Supplementary Data Sheet 2 — Medical assisted treatment clinic group survey. [file DataSheet2.pdf]

## Medical Assisted Treatment Clinic Group Survey

1. Gender:
   - ☐ Male
   - ☐ Female
   - ☐ Non-binary
   - ☐ Prefer not to answer

2. Age:
   - ☐ 18-24
   - ☐ 25-34
   - ☐ 35-44
   - ☐ 45-54
   - ☐ 55-64
   - ☐ 65 or over

3. Race/Ethnicity: (Select all that apply)
   - ☐ Asian/Pacific Islander
   - ☐ Black or African American
   - ☐ Hispanic/Latinx
   - ☐ Native American
   - ☐ White
   - ☐ Other:

4. What is the highest level of education you have attained?
   - ☐ Less than high school
   - ☐ High school or GED
   - ☐ Some college
   - ☐ College degree
   - ☐ Post college degree
   - ☐ Prefer not to answer

5. Overall would you say that you are content with your current situation.
   - ☐ Yes
   - ☐ No
   - ☐ Prefer not to say

6. Do you have a family history of alcohol use disorder?
   - ☐ Yes
   - ☐ No
   - ☐ Prefer not to answer

7. Do you have a family history of illegal substance use disorder?
   - ☐ Yes
   - ☐ No
   - ☐ Prefer not to answer

8. Do you have a family history of prescription substance use disorder?
   - ☐ Yes
   - ☐ No
   - ☐ Prefer not to answer

9. Do you have a history of depression?
   - ☐ Yes
   - ☐ No
   - ☐ Prefer not to answer

10. Do you have a history of ADD, OCD, bipolar disorder, or schizophrenia?
    - ☐ Yes
    - ☐ No
    - ☐ Prefer not to answer

11. As a child, where you forced to have a sexual experience?
    - ☐ Yes
    - ☐ No
    - ☐ Prefer not to answer

12. Do you have a personal history of alcohol use disorder?
    - ☐ Yes
    - ☐ No
    - ☐ Prefer not to answer

13. Do you have a personal history of illegal substance use disorder?
    - ☐ Yes
    - ☐ No
    - ☐ Prefer not to answer

14. Do you have a personal history of prescription substance use disorder?
    - ☐ Yes
    - ☐ No
    - ☐ Prefer not to answer

15. How old were you when you first had a drink of alcohol? (if you have never had a drink of alcohol skip to question 21).
    - ☐ <10
    - ☐ 11 – 15
    - ☐ 16 – 20
    - ☐ 21 – 30
    - ☐ 31 – 45
    - ☐ 46 – 65
    - ☐ >65

16. What type of alcohol was it?
    - ☐ Beer
    - ☐ Wine
    - ☐ Liquor

17. Who gave it to you?
    - ☐ Yourself
    - ☐ Parent
    - ☐ Grandparent
    - ☐ Other relative
    - ☐ Friend

18. Please check three feelings that best describe how alcohol made you ***feel*** when you first drank it?

- ☐ Euphoria
- ☐ Happiness
- ☐ Self-confident
- ☐ Well-being
- ☐ Normal
- ☐ Sick
- ☐ Depressed
- ☐ Sad
- ☐ Nothing

19. How long after your first experience with alcohol did you drink alcohol again?

- ☐ Immediately
- ☐ Days to weeks
- ☐ Weeks to months
- ☐ Years
- ☐ Don't remember

18. Have you ever been in treatment for alcohol use disorder? (if you answer no skip to #21)

- ☐ Yes
- ☐ No
- ☐ Prefer not to answer

19. In your own words, describe your motivations for drinking alcohol right before you entered your first treatment program for alcohol use disorder.

______________________________________________________________________________

______________________________________________________________________________

20. If you could name one factor that was the turning point in your recovery, what was it?

______________________________________________________________________________

______________________________________________________________________________

21. How old were you when you took an opioid for the first time? (If you have never taken an opioid for any reason you are done with this survey).

- ☐ <10
- ☐ 11 – 15
- ☐ 16 – 20
- ☐ 21 – 30
- ☐ 31 – 45
- ☐ 46 – 65
- ☐ >65

22.What medication was it?

- ☐ Codeine with Tylenol (Tylenol #3 or #4)
- ☐ Fentanyl (Actiq, Duragesic, Fentora, Abstral, Onsolis)
- ☐ Heroin
- ☐ Hydrocodone (Hysingla, Zohydro ER)
- ☐ Hydrocodone/acetaminophen (Lorcet, Lortab, Norco, Vicodin)
- ☐ Hydromorphone (Dilaudid, Exalgo)
- ☐ Meperidine (Demerol)
- ☐ Methadone (Dolophine, Methadose)
- ☐ Oxycodone (Roxicodone)
- ☐ Oxycodone/acetaminophen (Percocet)
- ☐ Other: ______________________________
- ☐ Don't remember

23. Who gave it to you?

- ☐ Physician
- ☐ Dentist
- ☐ Parent
- ☐ Relative
- ☐ Friend
- ☐ Acquaintance
- ☐ Other: _______________

24. For what reason did you take it?

_______________________________________________________________________________

25. Please check three feelings that best describe how the opioid made you ***feel*** when you first used it/them?

- ☐ Euphoria
- ☐ Happiness
- ☐ Self-confident
- ☐ Well-being
- ☐ Normal
- ☐ Numb
- ☐ Relief
- ☐ Accepted
- ☐ Strong
- ☐ Weak
- ☐ Loveable
- ☐ Good enough
- ☐ Sick
- ☐ Depressed
- ☐ Sad
- ☐ Nothing
- ☐ Focused

26. How long after your first experience with opioids did you use them again?

- ☐ Immediately
- ☐ Days to weeks
- ☐ Weeks to months
- ☐ Years
- ☐ Don't remember

27. In your own words, describe your motivations for using opioids right before you entered your first treatment program for opioid abuse.

_______________________________________________________________________________
_______________________________________________________________________________

28. If you could name one factor that was the turning point in your recovery, what was it?

_______________________________________________________________________________
_______________________________________________________________________________

Thank you for participating!
